# The Multiple Roles of Small-Angle Tilt Grain Boundaries in Annihilating Radiation Damage in SiC

**DOI:** 10.1038/srep42358

**Published:** 2017-02-09

**Authors:** Hao Jiang, Xing Wang, Izabela Szlufarska

**Affiliations:** 1 Department of Materials Science and Engineering, University of Wisconsin-Madison, WI, 53706, USA; 2 Department of Engineering Physics, University of Wisconsin-Madison, WI, 53706, USA.

## Abstract

Lattice defects generated by radiation damage can diffuse to grain boundaries (GBs) and be annihilated at GBs. However, the precise role of GBs in annihilating the segregated defects remains unclear. Here, we employed multi-scale models to determine how interstitials are annihilated at small-angle tilt GBs (STGBs) in SiC. First of all, we found the pipe diffusion of interstitials in STGBs is slower than bulk diffusion. This is because the increased interatomic distance at dislocation cores raises the migration barrier of interstitial dumbbells. Furthermore, we found both the annihilation of interstitials at jogs and jog nucleation from clusters are diffusion-controlled and can occur under off-stoichiometric interstitial fluxes. Finally, a dislocation line model is developed to predict the role of STGBs in annihilating radiation damage. This model includes defect flux to GBs, pipe diffusion in STGBs, and the interaction of defects with jogs. The model predicts the role of STGBs in annihilating defects depends on the rate of defects segregation to and diffusion along STGBs. STGBs mainly serve as diffusion channel for defects to reach other sinks when defect diffusivity is high at boundaries. When defect diffusivity is low, most of the defects segregated to STGBs are annihilated by dislocation climb.

Upon exposure to high-energy radiation environments (e.g., due to neutron, electron, or ion irradiations in nuclear reactors), point defects and clusters are generated in solids in amounts significantly exceeding their equilibrium concentrations. The accumulation of defects can lead to undesired consequences such as crystalline-to-amorphous transformation^1^, swelling^2^, and embrittlement^3^, and these phenomena can adversely affect the lifetime of components in nuclear reactors. It is known that interfaces, such as GBs, can act as defect sinks. This fact has inspired many studies that aim at improving radiation tolerance of materials by introducing a large number of internal interfaces.

While it is known that there is a strong thermodynamic driving force for defects to diffuse to GBs^4^,^5^,^6^, the questions of how GBs accommodate these defects and how these defects in turn change the sink ability of GBs remain unclear. Defect kinetics at interfaces is expected to have a significant impact on the ability of GBs to accommodate irradiation-induced defects, as shown for instance by Uberuaga *et al*.^5^. In this study the authors used object kinetic Monte Carlo models of defects at GBs, to show that the sink strength of GBs depends on the rate of in-boundary interstitial-vacancy mutual recombination, which is controlled by the diffusion of defects within the interface. The study by Uberuaga *et al*. assumed geometry of GBs to be a thin plate without atomic structures and therefore by construction this model did not account for such phenomena as the annihilation of defects at jogs on edge dislocations in STGBs^7^. The interplay between defect diffusion along GBs and defect annihilation at jogs may play an important role in determining the defect annihilation rate of GBs. In addition to point defects, defect clusters can also be present at GBs due to aggregation of point defects that had arrived at a GB^5^ or by direct formation of clusters in the displacement cascade that contains a GB^8^. The effect of defect clusters on the sink ability of GBs is still an open question.

SiC is a promising cladding and structural material for nuclear fission and fusion reactors due to its excellent mechanical strength, high-temperature stability and low neutron capture cross-section^9^,^10^,^11^. There have been several studies^12^,^13^,^14^,^15^,^16^,^17^,^18^ investigating the effect of grain refinement on the radiation response of SiC. However, the published trends have not been consistent throughout all these studies and they include reports of deterioration^12^,^13^,^14^,^16^, improvement^15^,^17^, and no change^18^ in radiation resistance of SiC with grain refinement. The discrepancies in experimental results further emphasize the importance of understanding the interplay between radiation induced defects and GBs. In this paper we investigate the kinetics of self-interstitials at STGBs and the impact of this kinetics on the long-term evolution of STGBs in irradiated SiC. In SiC it is well known that vacancies diffuse much slower (*ab initio* migration barriers of C and Si vacancies are 3.66 eV and 2.70 eV, respectively)^19^ as compared to interstitials (*ab initio* migration barriers of C and Si interstitials are 0.67 eV and 0.83 eV, respectively)^19^ and therefore the defect fluxes to GBs are dominated by interstitials^20^. However, the kinetics of interstitials and clusters in GBs in SiC has not been reported yet and it is not known how GBs transport and annihilate these defects. In addition, for multi-component materials, defect flux to GBs may be off-stoichiometric^20^ For instance, because the migration barrier of C interstitial (C_*i*_) is lower than that of Si interstitial (Si_*i*_), the flux of C_*i*_ to GBs is likely to be higher than that of Si_*i*_. It is unclear how the off-stoichiometric flux affects the sink strength of GBs. Understanding this effect may also be relevant for other multi-component ceramics (e.g., ZrC^21^) that are proposed as cladding and structural component in reactors.

## Results

### Annihilation of defects by diffusion along GBs to other sinks

**Figure 1:**
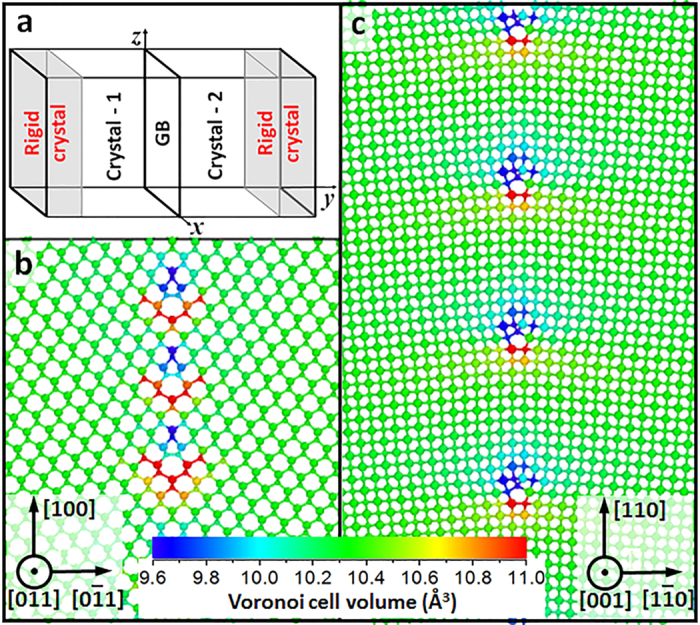
The simulation supercell and GB structures. (**a**) schematic drawing of the bicrystal supercell; (**b)** tilt axial view of [011] (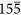), Σ73, *θ* = 13.4°; (**c)** tilt axial view of [001] (670), Σ85, *θ* = 8.8°. Si and C atoms are shown as large and small spheres, respectively. Atoms in panel (**b** and **c)** are colored by the Voronoi cell volume^37^. This volume is a measure of a local strain field.

Diffusion of defects along GBs to reach other sinks, such as surfaces and triple junctions, has been often considered an important pathway for annihilating defects in fine-grained materials^5^,^6^,^22^. In order to determine the diffusivities of interstitials in STGBs in SiC, we constructed six STGBs with the tilt axis along the [001] direction and three STGBs with the tilt axis along the [011] direction. The bi-crystal GB model used in this study is shown in Fig. 1a. The simulation supercell size is approximately 4 × 8 × 8 nm (x, y, and z in Fig. 1a). Molecular dynamics (MD) simulations are used to optimize atomic structure of the GBs and to relax stresses in all directions (see method section). The optimized structures of [001] and [011] STGBs consist of similar structural units as those previously found in diamond, cubic-Si, and SiC^23^,^24^,^25^. In Fig. 1b and c, respectively, we show two representative STGBs structures, [011] (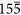) Σ73 and [001] (670) Σ85 (where Σ represents coincidental site lattice, […] represents the tilt axis, and (…) represents the GB plane). As expected, these STGBs are comprised of sets of edge dislocations at the interface. Detailed information (e.g., tilt angles, Σ value, and GB energies) of the 9 total STGBs used in this study can be found in Supplementary Fig. S1.

**Figure 2:**
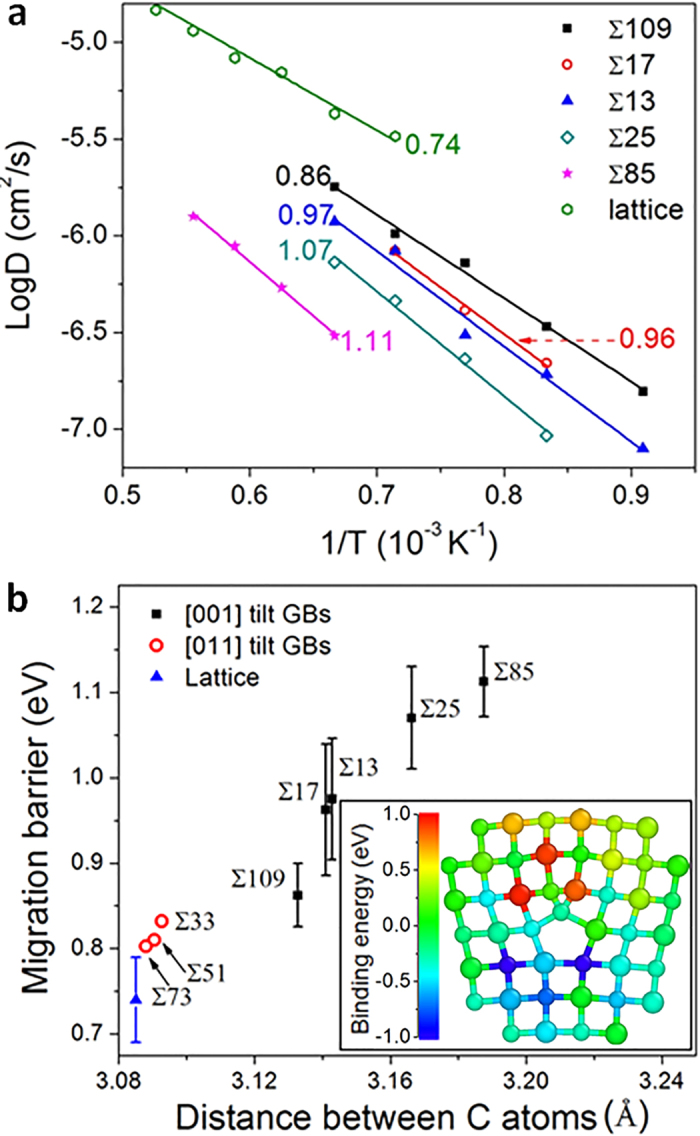
Diffusion coefficients and migration barriers of C_*i*_ at GBs. (**a)** Arrhenius plot of diffusion coefficients of C_*i*_ at [001] tilt GBs; numbers at the ends of each line represent the extrapolated migration barrier in eV; (**b)** The migration barrier as a function of distance between C atoms along the minimum energy pathway. The inset shows the binding energy of C_*i*_ forming a dumbbell at different lattice sites near a dislocation core in [001] Σ85 STGB. Negative binding energy (colored blue) means that the site is energy favorable for C_*i*_. Large spheres represent Si atoms, and small spheres represent C atoms.

We calculated diffusion coefficients for carbon (C_*i*_) and silicon (Si_*i*_) interstitials in STGBs (method section and supplementary materials). These diffusion coefficients are plotted as a function of temperature in Fig. 2a. By fitting the Arrhenius relationship to the data in Fig. 2a, we determined migration energy barriers, which are shown in Fig. 2b. Our calculations found that the diffusion coefficients of C_*i*_ in [001] STGBs are lower than in the bulk SiC by 1-2 orders of magnitude. Examination of C_*i*_ trajectories reveals that in [001] STGBs, C_*i*_ diffuses predominantly on a dislocation line along the tilt axis. The migration barrier of C_*i*_along the tilt axis (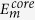) is in the range of 0.81–1.11 eV, which is higher than the migration barrier in bulk (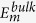 = 0.74 ± 0.05 eV, calculated using the same empirical potential)^26^. The increase in the migration barrier in [001] STGBs was validated for selected structures by density functional theory (DFT) calculations and the climbing image nudged elastic band method (details in supplementary materials). In [011] STGBs, C_*i*_ was also found to migrate primarily along the tilt axis and the migration barrier was found to be in the range of 0.80–0.83 eV. This value is slightly higher than the migration barrier in bulk SiC.

It is interesting to ask why the interstitial migration barriers in STGBs are higher than in the bulk, since GBs have been often considered to be pathways for fast transport of defects^27^,^28^,^29^. To understand the mechanisms underlying diffusion along GBs, in Fig. 2b we plot the migration barriers of C_*i*_ as a function of the distance between C lattice atoms along the migration pathway. Here, the distance between C lattice atoms is used because MD trajectories show that when C_*i*_ diffuses along the dislocation line in STGB, this defect always attaches itself to lattice C atoms to form dumbbells. As shown in Fig. 2b the migration barriers of C-C dumbbells increase monotonically with the distance between C lattice atoms along the migration path for both [001] and [011] STGBs. This trend means that indeed diffusion is controlled by the transition from one dumbbell configuration to another and this transition is in turn controlled by the distance between C atoms along the migration path. This observation is in contrast to typical models of interstitial diffusion, where a larger spacing between GB atoms provides more open space for transport of interstitials and leads to a lower migration barrier as compared to the bulk diffusion^30^. In the case of STGBs in SiC, although interstitials do segregate, as expected, to the tensile regions in the GBs (there is more free volume for the interstitial in these regions), the same tensile strain that lowers formation energy of interstitials at these sites, leads to an increase of migration barriers because of the increased distance between C atoms.

Our MD simulations found that in all STGBs considered in this study Si_*i*_ is immobile on the MD time scales (μs) at temperatures up to 1500 K. The lack of mobility of Si_*i*_ can be attributed to the local structural changes of GBs that take place when Si_*i*_ is incorporated (details are discussed in supplementary materials).

In addition to the diffusion of interstitials along the dislocation line, we have also investigated diffusion along the direction parallel to the tilt axis, but still within the GB (e.g., diffusion along the [110] direction in the [001] STGBs shown in Fig. 1c). Along this direction there are alternating regions of tensile and compressive strains; these strains arise due to the presence of edge dislocations that comprise the STGB. The tensile regions are energetically favorable for interstitials (because of the larger free volume) and there is a significant energy cost associated with the migration of the interstitials through the compressive regions. For instance, as shown in the inset of Fig. 2b, the energy of C_*i*_ increases by 2.0–2.5 eV when C_*i*_ moves from the tensile region (below the dislocation core) to the compressive region (above the dislocation core). It follows that the migration barrier is at least 2.0 eV. A similar lower bound on the migration barrier (1.6 eV) was found by us for the [011] STGBs. These barriers are much higher than the corresponding 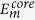 at each GB (Table S1 in supplementary materials). In addition, the high binding energies of interstitials to dislocation core (Table S1 in supplementary materials) make it unlikely for interstitials to leave the boundaries. These two factors explain the one-dimensional diffusion of interstitials along dislocation cores observed in our MD simulations.

### Annihilation of defect by jogs and jog nucleation

**Figure 3:**
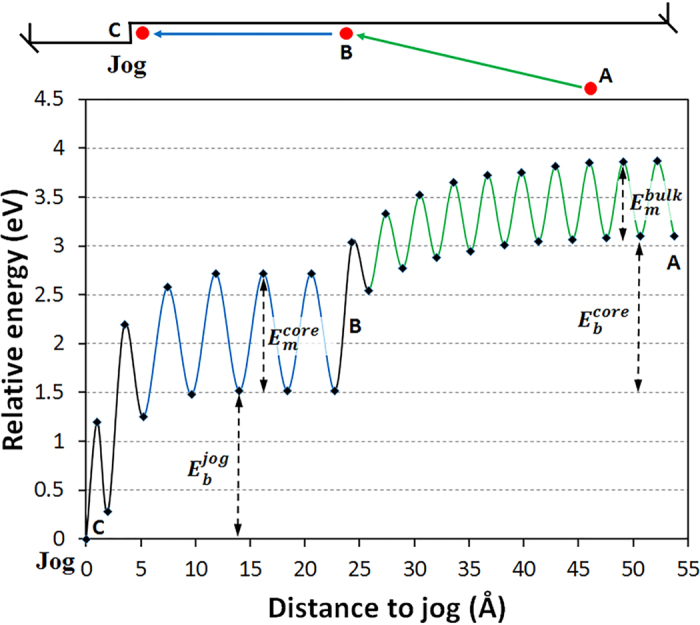
The energy landscape for annihilating of C_*i*_ at jogs. Position A represents C_*i*_ in the bulk, B represents C_*i*_ segregated to dislocation cores at STGBs, and C represents C_*i*_ attached to jogs. In the schematic drawing, the green straight line represents the migration of C_*i*_ from bulk SiC to the dislocation core, and the blue straight line represents the migration of C_*i*_ along the dislocation line to a jog. The corresponding parts of the energy landscape are labeled using the same color scheme. 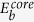 is the binding energy of C_*i*_ from bulk to dislocation cores, 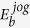 is the binding energy of C_*i*_ from dislocation core to jogs, 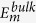 is the migration barrier of C_*i*_ in bulk SiC, and 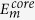 is the migration barrier of C_*i*_ at dislocation core along the dislocation line.

Interstitials arriving at a GB may bind to dislocation lines at pre-existing jogs, leading to dislocation climb. Annealing of defects at jogs involves multiple kinetic processes, including diffusion from bulk to dislocation cores, diffusion along dislocation cores to jogs, and attaching to jogs. It is important to ask whether annihilation of interstitials at jogs is limited by the diffusion of defects or by the reaction of attaching to jogs. In order to answer this question, in Fig. 3 we plot the energy landscape for C_*i*_ diffusion from bulk to a dislocation core and from the core to jogs in [001] Σ85. First, one C_*i*_ diffuses from bulk to dislocation cores with a migration barrier of 0.74 eV (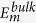). As C_*i*_ approaches GBs within a distance of 1 nm, the entire energy landscape as well as the transition barriers are lowered due to the interaction with dislocations^6^. The binding energy (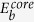) of C_*i*_ to dislocation core is approximately 1.6 eV. When C_*i*_ reaches a dislocation core, the migration barrier increases to 1.2 eV (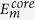) because of the stretched interatomic distance near the core, as discussed in the previous section. As C_*i*_ approaches the jog, the migration barrier starts to decrease at a distance of approximately 1 nm away from the jog. The activation energy barrier (~1 eV) for C_*i*_ attaching to a jog is lower than the migration barrier along the dislocation line (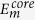
= 1.2 eV). Therefore, the annihilation of interstitials at jogs at STGBs of SiC can be considered to be a diffusion controlled process. Besides, the binding energy of C_*i*_ to jogs (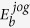) is as high as 1.5 eV so it is unlikely that once C_*i*_ is attached to a jog, it will be re-emitted and it will diffuse away from jogs. This binding energy is defined as the energy change when moving an interstitial from a jog to a ground-state configuration in bulk SiC.

**Figure 4:**
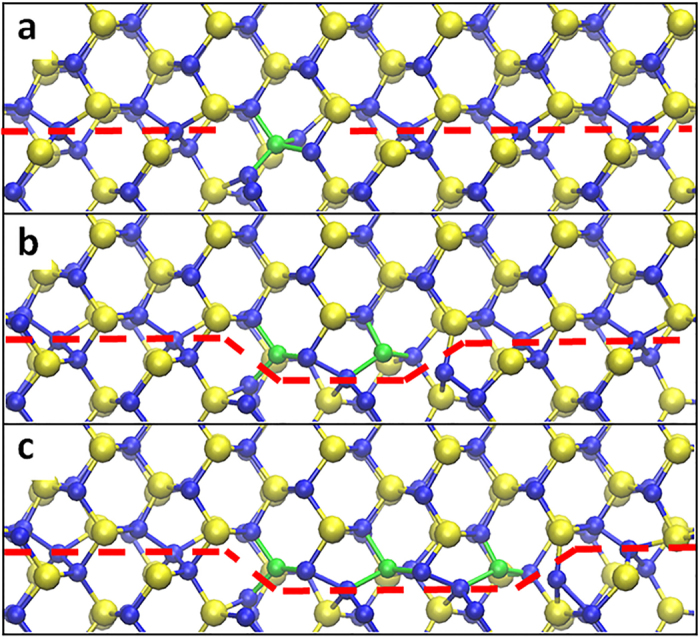
Nucleation process of a jog from C_*i*_ clusters. (**a)** A di-C_*i*_ cluster: the regular structure of dislocation line was disrupted at the site where the cluster was incorporated; (**b)** A jog nucleated from 4-C_*i*_ cluster; and (**c)**, Jog extension by loading two more C_*i*_ to the jog shown in (**b)**. The red dashed lines mark the position of the dislocation line. Jogs are present at the location where the dislocation line is shifted. Large yellow spheres represent Si atoms, and small blue spheres represent C atoms. Newly incorporated C atoms residing at Si sites are colored green. Newly incorporated C atoms residing at C sites are colored blue in the same fashion as other C atoms in the lattice.

Interstitials that have segregated to GBs can not only attach themselves to existing jogs, but they can also agglomerate into clusters^5^. Because of the high migration barriers of interstitial clusters in SiC^31^, annihilation of clusters by diffusion is limited. However, when a cluster is formed at STGBs, it can be annihilated by nucleating a new jog on a dislocation line. The activation energy for jog nucleation and the minimum cluster size required for nucleation are important parameters that determine the jog nucleation rate. In order to determine these parameters and to understand the mechanisms of jog nucleation, we conduct MD simulations at elevated temperatures. We first introduce a di-C_*i*_ cluster on the dislocation line and keep the system in equilibrium at 1250 K for 100 ns. The structure of di-C_*i*_ cluster after annihilating is shown in Fig. 4a. No diffusion or dissociation was observed in the simulation. Two more C_*i*_ were then loaded next to the di-C_*i*_ cluster at a distance of a few angstroms. The system was subsequently heated to 1250 K for 20 ns, followed by a quench to 0 K in 500 ps. In this process we found that a pair of jogs was nucleated from the 4-C_*i*_ cluster, as shown by the shift of the red line in Fig. 4b. We repeated the above simulations at different temperatures (the lowest temperature was 500 K) for both stoichiometric (the same number of C_*i*_ and Si_*i*_) and off-stoichiometric (C_*i*_only) clusters, and the nucleation of a jog pair was observed in all simulations within MD timescale (up to μs). We can estimate the upper bound for the reaction barrier of the nucleation process by assuming that one nucleation event took place at 500 K over the time of 1 μs. The upper bound estimate of the energy barrier is 0.66 eV, and it is lower than the migration barrier of C_*i*_ in bulk (0.74 eV) and in GBs (0.81–1.1 eV). This implies that the nucleation of jogs from interstitials clusters is a fast process and that rate of jog nucleation is controlled by diffusion of interstitials along the GB. In addition, our simulations show that nucleation of a jog in STGBs requires a cluster with sizes as small as four interstitials as seen in the simulations. The nucleated jog pair can further annihilate nearby interstitials, as shown in Fig. 4c where two C_*i*_ were loaded near the jog and annihilated at 1250 K within tens of ns.

The above analysis implies that a dislocation climb in STGBs can take place even if the flux of interstitials is off-stoichiometric. Since C interstitials can be incorporated into STGBs as antisites (C_Si_, shown as green spheres in Fig. 4), the atomic plane left behind a climbing dislocation is rich in C antisites. This is different from the climb mechanism proposed by Petroff and Kimerling^32^,^33^ for binary III-V semiconductors with a zinc blend crystal structure. According to their mechanism, if only one type of interstitials (e.g., of species A) diffuses to a jog in an alloy AB, then a Frenkel pair (an interstitial and a vacancy) of species B can nucleate near the jog. The interstitial of species B is then absorbed by the jog in order to keep the dislocation core stoichiometric and as the result the climbing dislocation leaves behind a vacancy rich dislocation plane. In contrast, in SiC, dislocation climb in STGBs does not require an equal number of C and Si atoms and it can take place by absorption of C_*i*_ only (leaving behind a plane rich in C_Si_). However, stoichiometry is still energy preferable in SiC. Our MD simulations found that if a Si_*i*_ is found next to a dislocation core or near a C-rich plane, it will undergo a reaction with C_*Si*_ (i.e., C_*Si*_ + Si_*i*_ → Si_*Si*_ + C_*i*_) to restore the local stoichiometry. This reaction has been found to be both thermodynamically favorable^19^,^34^ and kinetically feasible (i.e., with a barrier of 0.68 eV^19^) in bulk SiC.

To test the generality of our results, we repeated the above studies for other [001] and [011] STGBs and we found the same general processes of interstitial annihilating at jogs and jog nucleation from interstitial clusters (Supplementary Fig. S3). The following conclusions can therefore be made about defect annihilation in STGBs in SiC: (1) annihilation of defects at jogs is diffusion controlled (i.e., the energy barrier to defect attachment is lower than the barrier to diffusion); (2) jog nucleation from an existing interstitial cluster can be viewed effectively as a barrierless process (i.e., the nucleation barrier is lower than the migration energy); (3) the minimum number of interstitials needed to nucleate a jog pair is four, regardless of the cluster composition.

### Dislocation line model

**Figure 5:**
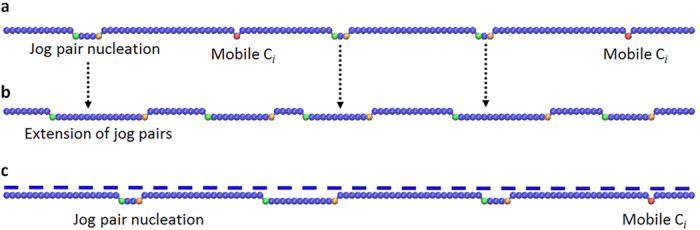
Snapshots from the dislocation line simulations. (**a)** Initial stage, where mobile C_*i*_ are loaded onto the line and diffuse to form clusters and nucleate jogs; (**b)** Extension of jog pairs by absorbing interstitials; and (**c)**, The entire dislocation line climbs down by a unit length, and new jogs nucleate on the line. Mobile C_*i*_ are colored red, jogs moving to the left are colored green, and jogs moving to the right are colored orange. The initial position of the dislocation line is shown by the straight dashed line in panel (**c**).

So far we have illustrated the details of kinetics of several processes that can annihilate interstitials in STGBs in SiC. These processes include pipe diffusion to other sinks, pipe diffusion to existing jogs, and jog nucleation from clusters. Here, we develop a dislocation line model that takes into account these processes as well as the flux of defects to GBs in order to predict how STGBs annihilate radiation-induced defects at long time scales. In this model, we use one single dislocation line to represent sets of edge dislocations at STGBs. This simplification is valid under two assumptions. First, each dislocation in a STGB annihilates defects independently. This assumption can be justified because of the high activation energy (over 1.6 eV) for interstitials to migrate between dislocations within STGBs, so there is no mass transport among dislocations. Second, all dislocations in one STGB behave similarly in response to uniform defect fluxes to GBs. Under this scenario, each dislocation receives similar numbers of interstitials from bulk and they climb at similar rates. The distance between neighboring dislocations is maintained and they will not run into each other. The length of the dislocation line is taken to be equal to the average grain diameter. The ends of the dislocation line are assumed to be ideal sinks for defects such as surface and triple junctions^5^,^35^. Once interstitials have diffused to either end of the dislocation line, they are annihilated by these sinks and therefore removed from the dislocation line. The flux of defects to the dislocation line is determined by the irradiation conditions (i.e., the dose rate, temperature, and grain size) and by the spacing between dislocation lines in a chosen STGB configuration. When interstitials reach a dislocation line, they can undergo one-dimensional diffusion along the dislocation line. They can also form clusters with other interstitials or be annihilated by attaching to existing jogs and clusters. Snapshots from simulations carried out using the dislocation line model are shown in Fig. 5. A detailed description of all assumptions, rates for different processes, and implementation of the dislocation line model is provided in the method section and in supplementary materials.

The flux of defects to GBs was calculated by using *ab initio* based rate theory model described in ref. 20. The rate theory model predicts that the flux of defects to GBs is dominated by interstitials, and that the ratio of the C_*i*_ flux to the Si_*i*_ flux (C/Si) to GBs ranges from 10 to 20, depending on the irradiation conditions. However, the rate theory model from ref. 20 ignores the possibility of defect clusters formation inside the grain, which assumption may potentially affect the stoichiometry of the flux. To take into account the uncertainties of the rate theory model, we treat the C/Si ratio as a parameter in our dislocation line model and we set it to be 1, 10, 20, and 100 to simulate cases of stoichiometric, as well as slightly, medium, and highly off-stoichiometric fluxes.

Simulations with the dislocation line model were performed for the following range of conditions: 1–5 displacement per atom (dpa) under irradiation conditions of 10^−5^–10^−3^ dpa/s, at temperatures of 200–1100 K, and with the grain diameters of 0.1–20 μm. This set of parameters covers most of the experimetnal conditions reported in studies on radiation resistance of nano- and microcrystalline SiC^12^,^13^,^14^,^15^,^16^,^17^,^18^. In addition, we developed a single parameter λ to represent interstitial accumulation rate at STGBs for various STGBs, grain sizes, and irradiation conditions. This parameter is defined as the interstitial diffusion distance divided by the dislocation line length (grain diameter). The interstitial diffusion distance is in turn defined as the average C_*i*_ diffusion distance along the dislocation line before the arrival of the next interstitial to any place on the dislocation line. It is calculated as (2*D*Δ*t*)^0.5^, where *D* is the diffusion coefficient of C_*i*_ along the dislocation line and *∆t* is the time interval between the arrivals of interstitials to the dislocation line. *∆t* is determined by the flux of interstitials to GBs, which in turn depends on the exact irradiation condition. A high value of λ implies a high chance for interstitials to diffuse to the ends of the dislocaiton line and a low chance to form clusters with the next interstitial arriving to GBs within the time interval *∆t*. Therefore, a high value of λ represents a low defect accumulation rate on the dislocation line. The values of λ of STGBs under various irradiation conditions are listed in Table S2 in supplementary materials.

**Figure 6:**
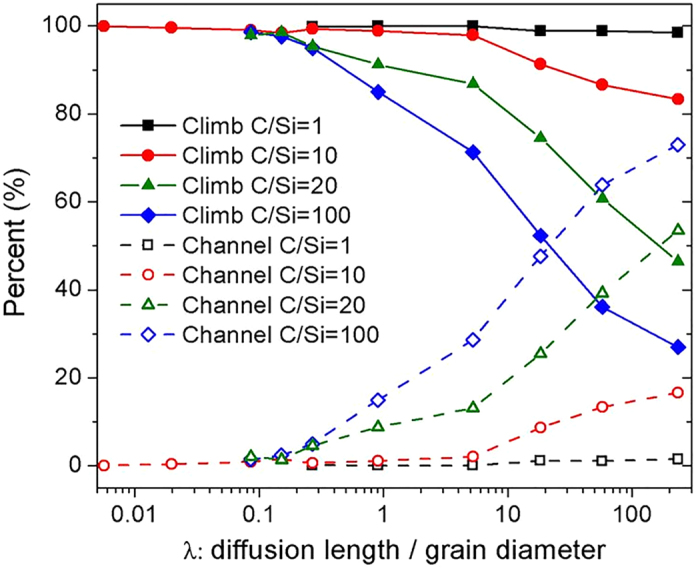
The role of STGBs in annihilating defects under various irradiation conditions. *Climb* means annealing of defects at jogs or by jog nucleation. *Channel* means diffusion of defects along GBs to other sinks. C/Si means the ratio of C_*i*_ to Si_*i*_that diffuse to GBs as defined in the main text.

When performing simulations with the dislocaiton line model, we count the number of interstitials that diffused to the ends of the dislocation line (e.g., surfaces and triple junctions), and the number that is annihilated by a dislocation climb (i.e., attaching to existing jogs and nucleating new jogs). We will refer to the regime when more than 50% of interstitials diffuse to other sinks along STGBs as a diffusion channel regime, and the regime when more than 50% are annihilated by dislocation climb as the climb regime. In Fig. 6, we plot the number of interstitials in the diffusion channel regime (labeled as channel in Fig. 6) and the dislocation climb regime (labeled as climb in the same figure) as a percentage of the total number of interstitials segregated to GBs. The horizontal axis corredsponds to the parameter λ, as defined previously.

Our dislocation line model predicts that the role of STGBs in annihilating radiation damage in SiC depends on the accumulation rate of interstitials at GBs, which is determined by the grain size and the irradiation conditions. For the case of an off-stoichiometric flux with C/Si = 20 or 100, there is a transition between the diffusion channel regime and the dislocation climb regime as a function of irradiation conditions and grain size. The diffusion channel regime occurs under conditions where the ratio λ is higher than 20. In general, this corresponds to cases (according to Table S2 in supplementary materials) of a relatively small grain (less than 100 nm in diameter) irradiated to a low dose rate (lower than 10^−3^ dpa/s) at a high temperature (higher than 800 K). Under these conditions, C_*i*_ that have segreated to GBs can diffuse quickly along dislocation lines to reach other sinks. This process allows C_*i*_ to avoid gettting trapped in immobile defect clusters along the dislocation line. The dislocation climb regime is found when a material has either a relatively large grain size (diameter larger than 100 nm), or it is irradiated to a high dose rate (10^−3^ dpa/s and higher), or it is irradated at relatively low temperatures (800 K and lower). Under these conditions more than 50% of C_*i*_ that diffused to STGBs are annihilated by dislocation climb. Immobile C_*i*_ clusters unavoidably form at GBs because of the high concentration of C_*i*_, a slow diffuion as well as the long diffusion distance required to reach other sinks. Interstitial clusters can nucleate jogs when cluster consists of at least four interstitials. An increase in the jog density at GBs further rises the fraction of interstitials that are annihilated by dislocation climb. The situation is qualitatively different for stoichiometric (C/Si = 1) and slightly off-stoichiometric fluxes (C/Si = 10), where we find STGBs mainly annihilate segregated interstitials by dislocation climb. In these cases, a high concentration of Si_*i*_ reached GBs and became immobilized on the dislocation line due to structural reconstruction discussed in supplementary materials These immobile Si_*i*_ can trap mobile Ci_*i*_ to form clusters, which in turn nucleate jogs and annihilate additional defects.

## Discussion and Conclusions

Defect clusters are usually considered to be detrimental to the thermomechanical properties of irradiated materials. As clusters are generally immobile, they cannot be easily annealed and their accumulation can lead to swelling, creep, and embrittlement. However, in STGBs in SiC, interstitial clusters can be beneficial as they can easily nucleate new jogs, which in turn serve as sinks for other defects and hence increase GBs’ sink ability. The ability of STGBs to transport point defects and to annihilate defect clusters implies a high sink strength of this type of boundaries. This conclusion is consistent with the experimental observation of an increased radiation resistance of nc-SiC grown by chemical vapor deposition (CVD)^15^,^17^. The CVD-grown nc-SiC samples have a texture and therefore contain a high concentration of low-angle GBs as compared to randomly oriented grains in a micro-crystalline samples^15^,^17^. Since studies on other types of nc-SiC samples have shown this material to have a lower resistance to radiation-induced amorphization than the microcrystalline SiC^15^,^17^, it is possible that the STGBs present in CVD samples contribute to their increased radiation resistance. Other phenomena proposed to be responsible for the increased resistance of CVD grown nc-SiC samples include the presence of stacking faults in these samples^17^,^36^ and the long-range stress-field of STGBs that increases the defect flux to STGBs^6^ in SiC.

Our dislocation line model can also provide information about the rate of dislocation climb in STGBs under irradiation. We found the climb rate to be sensitive to the grain size. For μc-SiC (e.g., grain diameter ~1 μm), the climb rate is less than 1 atomic spacing (~0.2 nm) per dpa in the dislocation climb regime. For nc-SiC (e.g., grain diameter ~50 nm) in the dislcoation climb regime, the climb rate can be as large as 5 atomic spacings (~1 nm) per dpa. Because dislocation lines at STGBs in larger grains are longer, they require higher numbers of interstitials to attach in order for the entire dislocation to climb one unit atomic spacing. Therefore, dislocation lines in STGBs in larger grains have a low climb rate per dpa as compared to their conterparts in smaller grains. The high dislocation climb rate at STGBs in nc-SiC implies GBs undergo significant structural evolutions under irradiation. For instance, dislocation cores can climb to triple junctions or surfaces and be annihilated there. In the meantime, new dislocation cores can nucleate at the boundaries to maintain the grain misorientation. However, this is unlikely to happen in μc-SiC because of the low climb rate.

In summary, we found that once interstitials have segregated to the STGBs, the pipe diffusion of these defects in these GBs is slower than bulk diffusion. This is because the stretched interatomic distance at dislocation cores raises the migration barrier of interstitial dumbbells. Furthermore, we found that the annihilation of interstitials at jogs has a low activation energy and thus is a diffusion-controlled process. Jog nucleation from interstitial clusters is also found to be a process with a very low activation energy (relatively to the migration energy barriers) when the cluster size is equal to or larger than four, regardless of its composition. Finally, a dislocation line model was developed to take into account defect flux to GBs, pipe diffusion of defects in STGBs, and interactions between defects and jogs. This model reveals the multiple roles of STGBs in annihilating radiation-induced defects in SiC. It predicts that STGBs mainly serve as a diffusion channel for defects to reach other sinks like surface when the defect accumulation rate at boundaries is low. The diffusion channel regime occurs in nano-crystalline materials with small grain diameter (smaller than 100 nm) irradiated under low dose rate (lower than 10^−3^ dpa/s) at high temperature (higher than 873 K). In other cases, when the accumulation rate is high, most of the defects diffused to STGBs are annihilated by dislocation climb.

## Methods

### Grain boundary structure

[001] and [011] STGBs with tilt angles smaller than 35° were generated in a bicrystal supercell. When we attach two grains together to create an interface, some atoms might be too close to each other. Therefore if two atoms were found at a distance smaller than 1.9 Å, one of them was removed. When deciding which atom to delete, we applied a rule that we maintain C-Si bonds across the interface and avoid energetically less favorable homonuclear bonds (C-C or Si-Si). We generated structures with both stoichiometric and off-stoichiometric dislocation cores (either with an excess of C or Si atoms) in order to find a configuration with the lowest GB energy. A schematic drawing of the supercell is shown in Fig. 1a. The size of the supercell is approximately 8.0 nm × 3.5 nm × 9.1 nm along *x, y*, and *z* directions, respectively. We apply periodic boundary conditions in the directions parallel to the boundary plane (*x* and *z* axes). We make the outermost 4 layers of atoms normal to the *y* axis as rigid. The rigid slabs are allowed to move along the *y* direction to relax stress, and the length of the system along *y* direction is large enough to avoid unphysical interactions between GBs and the rigid slabs. To find the energetically most favorable configurations, we first perform simulation in a constant volume, constant temperature (NVT) ensemble at T = 1000 K for 20 ns with a time step of 1fs. The system was then quenched to 0 K in 50 ps in constant temperature and pressure (NPT) ensemble for 20 ns with a time step of 0.5 fs to ensure the external pressure was relaxed to zero. Finally, we use the conjugate gradient method to minimize the system energy and we calculate GB energy *E*_GB_ as


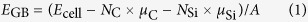


Here, *E*_*cell*_ is the energy of the supercell, and *N*_*C*_ and *N*_*Si*_ are the numbers of C and Si atoms, respectively. *μ*_*C*_ and *μ*_*Si*_ are the chemical potentials for C and Si atoms, respectively, and we choose *μ*_*C*_ = *μ*_*Si*_ = 0.5 × *μ*_*SiC*_ where *μ*_*SiC*_ is the energy of bulk 3C-SiC. For [001] and [011] STGBs, we found that boundaries with excess C atoms in the core (Fig. 1b and c) have the lowest GB energy. Therefore, we use the boundaries with excess C atoms throughout our study. The optimized GB structures of [001] and [011] STGBs have similar structural units as the equivalent GBs in diamond, cubic-Si, and SiC^23^,^24^,^25^. The Gao-Weber potential^34^ is used in our MD simulations. This potential was chosen here not only because it can correctly describe GB structure, but also because it can describe the kinetics of intrinsic defects with a reasonable agreement with first principle calculations. For instance, the migration barrier of C interstitials in bulk is 0.74 ± 0.05 eV according to Gao-Weber potential^26^ and 0.67 eV according to DFT^19^.

### Defect ground state (GS) identification

First, a single interstitial was loaded on one dislocation line in the GB plane. The system was heated to 1250 K in 5 ns and then quenched to 0 K in 5 ns with a time step of 0.01 fs in NVT ensemble using Gao-Weber potential^34^. We performed on the average 3 heat-quench cycles for each defect and each GB. Snapshots of the simulations were saved every 5 ps (producing ~2,000 snapshots per one heat-quench cycle) and each of the saved configurations was relaxed at 0 K using the conjugate gradient method. The binding energy *E*_b_ (or the segregation energy) of interstitials in each relaxed configuration was calculated as


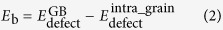


where 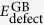 is the energy of the bicrystal supercell with a defect at GB, 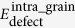 is the energy of the same bicrystal supercell with a defect far away from the GB (in the crystalline region). The lowest *E*_b_ configurations are taken to be the ground states of C_*i*_ and Si_*i*_ in [001] and [011] STGBs. Binding energies of C and Si interstitial to [001] and [011] tilt GBs are listed in Table S1 in the supplementary materials.

### Diffusivity and migration barrier calculations

An interstitial was first loaded at GBs in its GS configuration and then the system was heated to a chosen temperature and equilibrated there in NVT ensemble for 50 ns using classical MD. The diffusion coefficient *D* can be determined from the mean squared displacements of all *N* atoms in the simulation cell


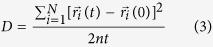


where *n* is the dimensionality of the diffusion (here *n* = 1 because interstitial diffuse along the tilt axis), *t* is the simulation time, 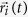 is the position of the *i*-th atom at time *t*, and 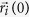 is its position at *t = 0*. In the calculations of diffusion, the dynamics was monitored for over 20 ns and *D* was averaged over the results from five independent simulations performed at each temperature. The migration barrier *E*_*m*_ was calculated from *D* based on the Arrhenius relation


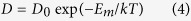


where *D*_*0*_ is the pre-exponential factor, *k* is the Boltzmann constant, and *T* is the temperature. For the case [001] STGBs, interstitial diffuses one-dimensionally along the dislocation line, so *n* is set as 1 in equation 3. However, because the binding energies of C_*i*_to [011] GBs are relatively low (~1.1 eV, Table S1 in supplementary materials), interstitial re-emission was occasionally observed within the simulation timescale for [011] GBs. In this case, equation 3 gives a mixed diffusivity of diffusion along GB and bulk diffusion near the interface. Therefore, equations 3 and 4 are not applicable for [011] GBs if we want to determine the diffusion barrier along the dislocation line. In order to determine such barriers, climbing image nudged elastic band calculations with Gao-Weber potential^34^ were conducted along several paths that were frequently observed in MD simulations, and the one with the lowest barrier was taken to be the reported migration barrier. Migration barriers of C interstitials in [001] and [011] tilt GBs along the dislocation line are listed in Table S1 in the supplementary materials.

### The dislocation line model

The model is based on the following assumptions, which are developed based on observations from MD simulations of STGBs in SiC:

1. C_*i*_ performs one dimensional diffusion along the dislocation line with a barrier that is different for each STGBs. However, we found that when predicting the role of GBs in annihilating defects, it is not critical to choose a specific GB and the corresponding migration barrier. This is because the definition of diffusion length to dislocation length (parameter λ), contains information about defect kinetic parameters, including the migration barrier. This is illustrated in details in supplementary materials section 6.
2. Movement of a jog by a unit distance requires attachment of 2 interstitials to the jog. This is due to the nature of dislocations in SiC. Specifically, as shown in Fig. 1b and c, the dislocation plane is composed of 2 atomic layers. If a pair of C_*i*_ or Si_*i*_ is attached to the jog, no antisite is produced as the jog moves. If two C_*i*_ are attached to the jog, a C antisite is produced during the movement of the jog.
3. Once formed, C-rich core and C-rich half plane are stable in the system.
4. Si_*i*_ does not diffuse along GB, but it can restore the stoichiometry of a C-rich site (if it diffused to such a site from bulk SiC). A mobile C_*i*_ is released during this process.
5. Clusters are immobile, and can nucleate a jog when the cluster size is equal to or larger than four and this is a barrierless process.
6. Interstitial re-emission to bulk was ignored for the following reasons. We can calculate the rate 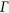 of an event as 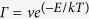 where *v* is vibrational frequency of atoms, *E* is the activation energy barrier, *k* is the Boltzmann constant, and *T* is temperature. In the temperature range of interest (500–1000 K), we found that the hop rate during diffusion is 2–3 orders of magnitude higher than the re-emission rate (migration barriers and binding energies are listed in Table S1 in supplementary materials). Therefore, it is reasonable to assume that diffusion dominates the kinetics of interstitials at STGBs.
7. Ends of the dislocation line are ideal sinks, such as surfaces and triple-junctions. This assumption has been often used for GB models such as those reported in literatures^5^,^35^.

The model was developed in the following steps. First, the flux *J* of interstitials to STGBs was calculated from the rate theory model reported in ref. 20. *J* is in the units of #/(m^2^ × s) and the fluxes under various irradiation conditions are listed in Table S2 in the supplementary materials. Given the spacing *d* between dislocation lines in a given STGB, the flux to dislocation line can be approximated as *J* × *d* (in the units of #/(m × s)). The length of a dislocation line *L* in STGB was assumed to be equal to the grain diameter, and then the time interval *∆t* to load one interstitial to dislocation line was calculated as ∆*t* = (*J* × *d* × *L*)^−1^ in the units of seconds. That means that one interstitial is loaded to the dislocation line every *∆t* seconds, and the predefined ratio of C_*i*_ flux to Si_*i*_ interstitial flux to GBs determines whether a C_*i*_ or Si_*i*_ is loaded. In the meantime, mobile C_*i*_ at GBs diffuse one dimensionally along the dislocation line by a distance of (2*Dt*_step_)^0.5^, where (2*Dt*_step_)^0.5^ is the average diffusion distance of C_*i*_ within a single time step of *t*_step_ (<∆*t*). After the diffusion of C_*i*_ within a small time step of *t*_step_, an algorithm was implemented to check conditions for events such as cluster formation, annihilation of interstitials at jogs and dislocation ends, jog nucleation, etc. The criteria to determine whether these events can occur is detailed in supplementary materials section 5. The simulation clock was advanced by *t*_step_ in each step in the dislocation line model. Within each time step, events such as loading interstitials to the dislocation line, diffusion of interstitials, and annihilation of interstitials to jogs, etc., are considered. A pseudo-code that shows the implementation of the model is shown in Fig. S5 in supplementary materials. Snapshots from the dislocation line simulations are shown in Fig. 5 and a movie of a full simulation can be found in supplementary video 1.

## Additional Information

**How to cite this article**: Jiang, H. *et al*. The Multiple Roles of Small-Angle Tilt Grain Boundaries in Annihilating Radiation Damage in SiC. *Sci. Rep.* **7**, 42358; doi: 10.1038/srep42358 (2017).

**Publisher's note:** Springer Nature remains neutral with regard to jurisdictional claims in published maps and institutional affiliations.

## Supplementary Material

Supplementary Materials

Supplementary Video 1
