# Supplementary material for: The Multiple Roles of Small-Angle Tilt Grain Boundaries in Annihilating Radiation Damage in SiC
Source: Sci Rep. 2017 Feb 9;7:42358. doi: 10.1038/srep42358 (PMC5299421; doi:10.1038/srep42358)
Supplement: Supplementary Materials [file srep42358-s1.doc]

**Supplementary materials for**

**The Multiple Roles of Small-Angle Tilt Grain Boundaries in Annihilating Radiation Damage in SiC**

Hao Jiang^1^, Xing Wang^2^, *Izabela Szlufarska^1,2^

^1^Department of Materials Science and Engineering, ^2^Department of Engineering Physics

University of Wisconsin-Madison, WI, 53706

**Properties of all [001] and [011] STGBs studied in this paper**


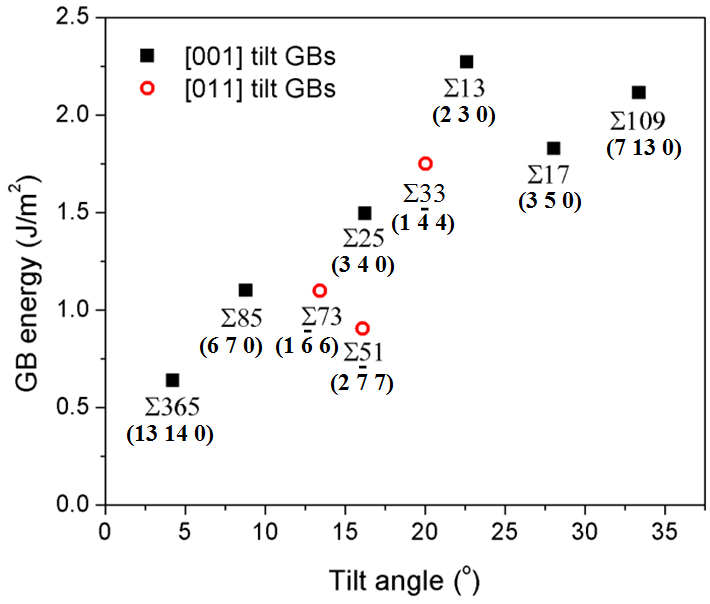


**Figure S1** | Energies of [001] and [011] tilt GBs as a function of tilt angle. The label by each symbol represents the coincidental site lattice and median plane of each GB.

**Table S1** | Binding energies and migration barriers of C and Si interstitials in [001] and [011] STGBs in 3C-SiC, as calculated by Gao-Weber potential^1^. Binding energy is defined by Eq. (2) in the main text.

| Grain Boundary | C interstitial | | Si interstitial |
| --- | --- | --- | --- |
|  | Binding (eV) | Migration(eV) | Binding (eV) |
| [001]Σ85 | -1.60 | 1.11±0.04 | 2.38 |
| [001]Σ25 | -1.52 | 1.07±0.04 | 2.35 |
| [001]Σ13 | -1.44 | 0.97±0.07 | 2.31 |
| [001]Σ17 | -1.41 | 0.96±0.08 | 2.29 |
| [001]Σ109 | -1.36 | 0.86±0.04 | 2.21 |
| [011]Σ33 | -1.16 | 0.83 | 1.81 |
| [011]Σ51 | -1.13 | 0.81 | 1.75 |
| [011]Σ73 | -1.11 | 0.80 | 1.71 |

**Ground state of interstitials in [001] and [011] STGBs**

**
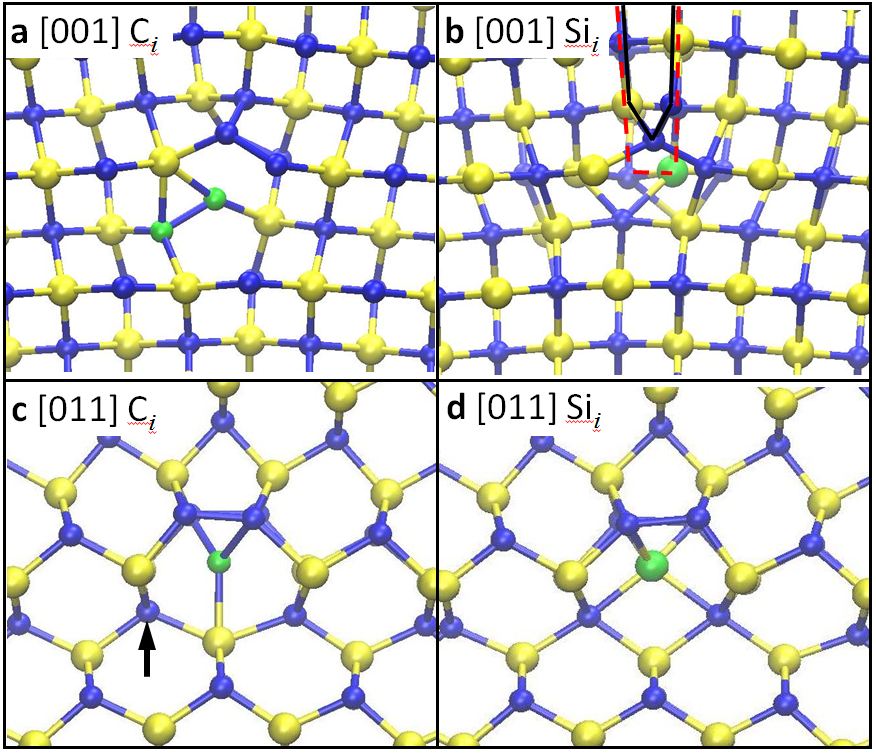
**

**Figure S2** | GS structures of interstitials in [001] and [011] tilt GBs. **a**, C*_i_* in [001] Σ85; **b**, Si*_i_* in [001] Σ85; **c**, C*_i_* in [011] Σ73; and **d**, Si*_i_* in [011] Σ73. Interstitials are colored green. Large spheres are Si atoms and small spheres are C atoms. In **a** two C atoms are colored green (although there is only one interstitial) because of the dumbbell configuration. The black solid lines in **b** enclose a dislocation plane terminated by one C atom. The red dashed lines in **b** enclose a dislocation plane terminated by one Si and one C atom. This latter configuration was formed through reconstruction of the dislocation core that followed absorption of one Si*_i_*. Atoms in the layer marked by red dashed lines are further away from reader than the layer marked by black solid lines, which is also reflected by lighter colors. The black arrow in panel **c** labels the C lattice site where the interstitial (green) will move to and form a dumbbell as the interstitial migrates through the lattice.

The ground states (GS) of C*_i_* and Si*_i_* at STGBs are identified by molecular statistics and are shown in Fig. S2. The GS of C*_i_* at [001] STGB is a dumbbell configuration (Fig. S2a), which is similar to the GS of this defect in a perfect SiC lattice^1,2^. In contrast, Si*_i_* is incorporated into the dislocation core by structural reconstruction (Fig. S2b). The dislocation plane was initially C-terminated and it transformed into a SiC-terminated plan (indicated by the red dashed lines in Fig. 2b) upon loading of a Si*_i_*. For [011] Σ73, the GSs of both C*_i_* and Si*_i_* are similar to each other and they are shown in Figs. S2c and S2d. The interstitial resides at the center of the largest free volume at the dislocation line. This interstitial is bonded to neighboring C atoms in the configuration that resembles a local atomic environment in a perfect SiC lattice.

**DFT-CINEB calculation of the migration barriers of C*_i_* at [001] Σ85**

In order to confirm predictions from classical MD simulations that the migration barrier of C*_i_* in STGBs is higher than in bulk, Density functional theory (DFT) simulations were performed using the Vienna Ab Initio Simulation Package (VASP)^3^. The electron-ion interactions are described by the projector-augmented wave (PAW) method. We use PAW pseudopotentials with the valence electron configuration of 2s^2^, 2p^2^ for C and 3s^2^, 3p^2^ for Si. The cut-off energy for plane-wave basis is set at 500 eV. The gamma point is used to sample the Brillouin zone. To obtain the migration barrier of C*_i_* in [001] Σ85 GB, we first constructed a supercell of the interface with one dislocation core in the center of the cell, as shown in Fig. S2a. The size of the cell is 1.3 nm × 1.3 nm × 3.2 nm, and it contains 552 atoms. Regions between the red and black dashed lines are frozen to prevent reconstruction because of un-physical interactions with the periodic images under periodic boundary conditions. The supercell was relaxed using a force convergence threshold of 0.02 eV/Å. We then perform Climbing Image Nudged Elastic Band (CI-NEB) calculation^4^ to determine the migration barrier of C*_i_* along the lowest barrier migration pathway identified by empirical Gao-Weber potential^1^. Along this path way, C*_i_* first hop from the ground state (dumbbell colored green in Fig. S2a) to a neighboring C lattice site (labeled by the black arrow in the same figure) to form a dumbbell at this site. Then the C*_i_* migrates from this site to another neighboring lattice site (labeled by the red arrow in Fig. S3a) where it forms a ground state configuration. One intermediate image is used for each hop in the CI-NEB calculations and the force convergence threshold is set at 0.05 eV/Å. The calculated energy landscape along the minimum barrier migration path is shown in Fig. S3b. One can see that the energy landscapes from DFT and from GW potential are qualitatively similar. In addition, the migration barrier of C*_i_* in GBs (1.3 eV) is higher than the diffusion barrier in bulk (0.67 eV)^5^. The higher migration barrier in GBs confirms that C*_i_* diffusion is slowed down in STGBs compared to bulk diffusion.


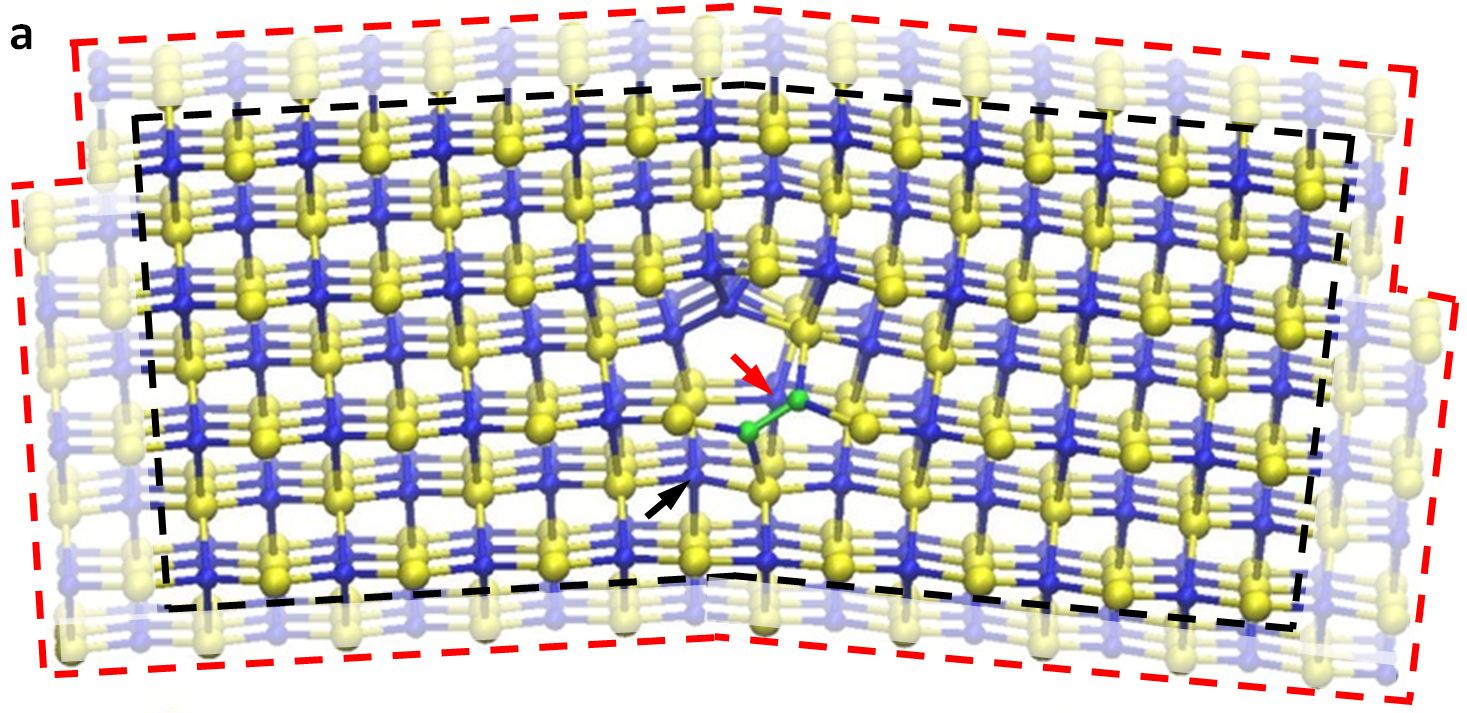


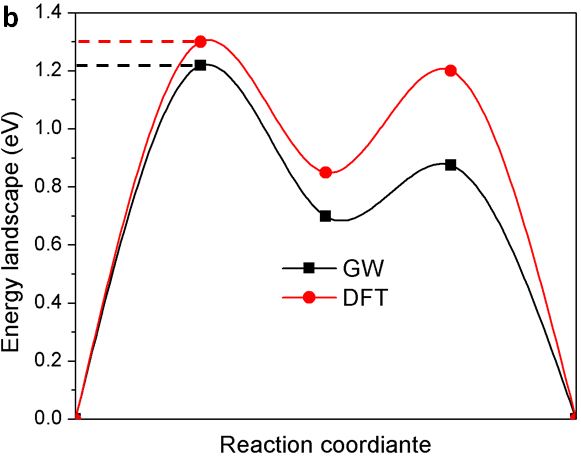


**Figure S3** |DFT calculations of the migration barrier of C*_i_* in [001] Σ85 STGB. **a**, Atomic configuration showing the ground state of C*_i_* where the dumbbell structure is colored green. Atoms between the red and black dashed lines are frozen in DFT calculations. Starting from this initial state, the interstitial diffuses to the site marked by the black arrow and forms a dumbbell. It then diffuses to the site labeled by the red arrow and forms a dumbbell, which is a symmetry equivalent configuration to the initial configuration. **b**, the energy landscape along the diffusion pathway obtained from DFT and Gao-Weber potential using a supercell with the same number of atoms.

**Jog nucleation from interstitial clusters in [011] Σ73**

**
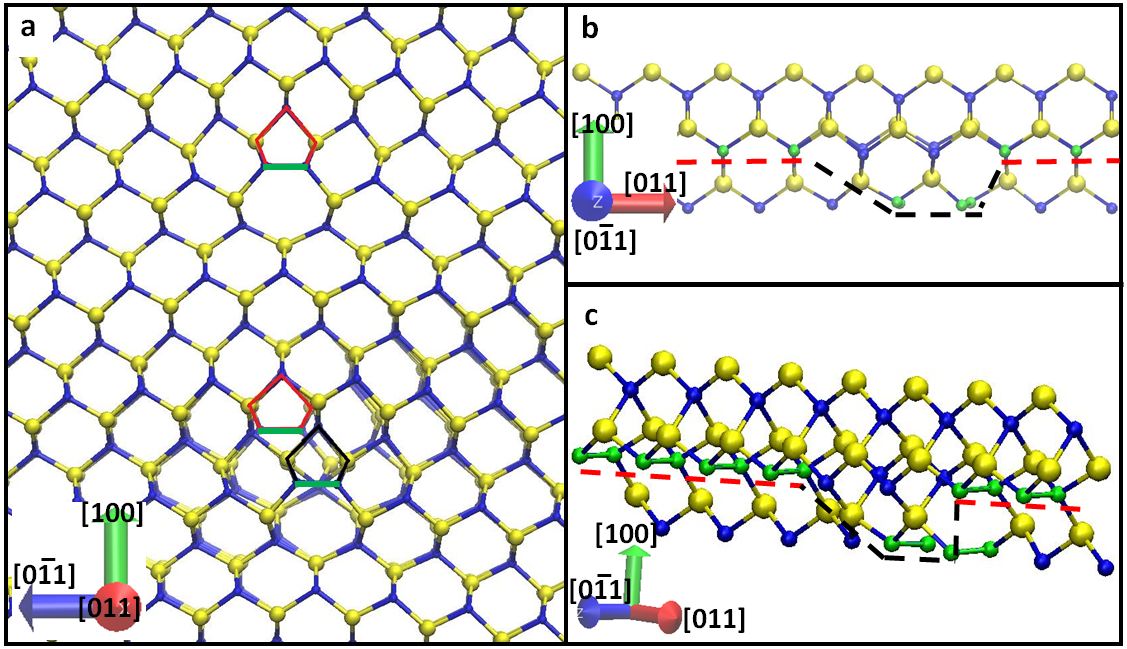
**

**Figure S4** | Jog nucleation from small interstitial clusters in [011] Σ73 STGB. **a,** A view of the boundary along the tilt [011] axis after loading two pairs of C*_i_* and Si*_i_*. Pristine dislocation core is colored red and the black color marks the section of the dislocation line that performed a climb during jog nucleation. Carbon-carbon bonds at dislocation cores are colored green. **b**, A view of the dislocation core structure along the [
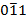
] axis. **c**, A view of the dislocation core structure in a tilted perspective. A red dashed line represents the position of the dislocation line without jogs, and the black dashed line represents the nucleated jog pair. Large yellow spheres represent Si atoms, small blue spheres represent C atoms, and small green spheres represent C atoms in the dislocation core.

In the main text, we have reported MD simulations of jog nucleation from defect clusters in [001] STGBs. We found that on MD time scales jog nucleation can happen at low temperature (as low as 500 K). To test the generality of the fast jog nucleation process, here we conduct analogous MD simulations for [011] Σ73 STGB. A pair of C*_i_* and Si*_i_* was first loaded onto the dislocation line to form a di-interstitial cluster. Two more interstitials (one C*_i_* and Si*_i_*) were then loaded next to the di-interstitial cluster at a distance of a few angstroms. The system was subsequently heated to 500 K for 0.2 μs, followed by a quench to 0 K in 500 ps. Similarly to the case in [001] STGBs shown in the main text, here we observed the nucleation of a jog pair from a four-interstitial cluster in [011] STGBs (see Fig. S3). To further test whether off-stoichiometric clusters can nucleate jogs, we repeated the above simulation procedure for a 4-C*_i_* cluster. We found clusters that are rich in C*_i_* can also nucleate jogs in [011] STGBs at an expense of C antisite formation. Therefore, based on MD simulations of jog nucleation in both [001] and [011] STGBs, we show that in SiC jog nucleation from an existing interstitial cluster on dislocation line can be viewed as diffusion-controlled process. Besides, we found the minimum number of interstitials needed to nucleate a jog pair to be four, regardless of the composition of the cluster.

**Pseudo-code for the dislocation line model**


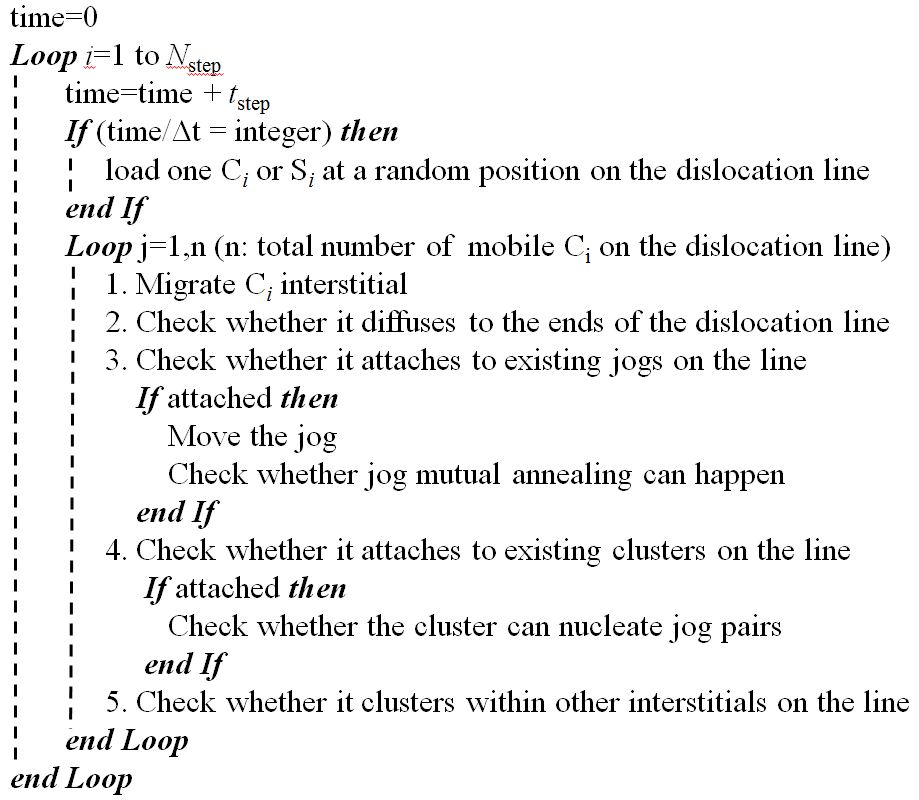


**Figure S5** | Pseud-code for the dislocation line model.

A pseudo-code of the dislocation line model is shown in Fig. S5. Starting from time equal to zero, we advance the simulation clock by a time step of *t_step_*. ∆*t* is the time interval between arrivals of interstitials to the dislocation core, which is determined by the rate theory model and illustrated in the method section of the main paper. To maintain an accurate description of defect kinetics, here we set *t_step_* to be 1/10 of the minimum ∆*t* calculated for all chosen radiation conditions and grain sizes. The minimum ∆*t* is 0.1s (see Table. S2) so *t_step_* is 0.01s.

At the beginning of each time step, we first check whether an interstitial should be loaded to the dislocation line. The loading process corresponds to the diffusion of interstitials from bulk to STGBs. Every ∆*t* (time/Δt = integer in the pseudo-code), one interstitial is loaded on the dislocation line at a random position. The predefined ratio of C*_i_* flux to Si*_i_* flux (C/Si) to GBs determines if the loaded interstitial is a C*_i_* or Si*_i_*. We then check and execute all kinetic process that can occur on the dislocation line. These kinetic process are labeled from 1 to 5 in Fig. S5 and we below we describe them in detail one by one.

The first process is the diffusion of a mobile C*_i_* along the dislocation line. C interstitials that are not attached to any clusters or jogs are considered as mobile. Each mobile C*_i_* diffuses a distance of (2*Dt*_step_)^0.5^ in each time step. (2*Dt*_step_)^0.5^ is the average diffusion distance of C*_i_* along the dislocation line within *t*_step_.

The process labeled as 2 in Fig. S5 is the annihilation of interstitials by other sinks at the ends of the dislocation line. In the model, if a C*_i_* is within a distance of (2*Dt*_step_)^0.5^ to either end of the dislocation line, it is removed from the system. The threshold value of (2*Dt*_step_)^0.5^ is used because if a C*_i_* is within such a distance to either end, it can diffuse to the end within *t*_step_, the time step in the model. The same distance threshold value is used in the following processes.

The process labeled as 3 in Fig. S5 is the attachment of interstitials to existing jogs on the dislocation line. In the model, if a C*_i_* is within a distance of (2*Dt*_step_)^0.5^ to a jog, it attaches to the jog. A jog will move by one unit distance whenever two interstitials attach to it. If two jogs move in the opposite directions and meet each other, they mutually annihilate.

The process labeled as 4 in Fig. S5 is the attachment of interstitials to existing clusters onto the dislocation line. In the model, if a C*_i_* is within a distance of (2*Dt*_step_)^0.5^ to a cluster, it attaches to the cluster. The number of interstitials in the cluster increases by 1 because of the attachment. In addition, a cluster will transfer to a jog pair when the number of interstitials in the cluster is equal or larger than 4.

The process labeled as 5 in Fig. S5 is the formation of clusters on the dislocation line. In the model, if one C*_i_* is within a distance of (2*Dt*_step_)^0.5^ to another interstitials (C*_i_* or Si*_i_*), they form an immobile cluster. If one of the interstitials is Si*_i_*, the position of the formed cluster is the position of the Si*_i_*. This is because Si*_i_* is immobile along the dislocation core and C*_i_* diffuses toward the Si*_i_* to form a cluster. If both of the two interstitials are C*_i_*, the position of the formed clusters is at the center of the distance between the two C*_i_* as they diffuse toward each other to form a cluster.

**Table S2** | List of irradiation conditions, grain sizes, and the total interstitial fluxes to GBs as predicted by the rate theory model. Values of the parameter λ, defined as the diffusion distance divided by the dislocation length (see main text for details), for [001] Σ85 and [011] Σ79 under various conditions are listed in the last two columns.

| Dose rate | Grain diameter | Temp. | Interstitial flux to GB | [001] Σ85  λ | [011] Σ79  λ |
| --- | --- | --- | --- | --- | --- |
| (dpa/s) | (μm) | (K) | (#/m^2^s) | (unitless) | (unitless) |
| 6.5×10^-5^ | 10 | 873 | 6.68×10^14^ | 0.01 | 0.04 |
| 6.5×10^-3^ | 1 | 750 | 4.98×10^15^ | 0.02 | 0.20 |
| 6.5×10^-3^ | 0.1 | 573 | 6.00×10^15^ | 0.04 | 0.86 |
| 6.5×10^-3^ | 1 | 873 | 2.00×10^15^ | 0.09 | 0.90 |
| 6.5×10^-3^ | 0.1 | 573 | 1.09×10^15^ | 0.10 | 2.00 |
| 6.5×10^-5^ | 10 | 1173 | 4.36×10^13^ | 0.14 | 0.63 |
| 6.5×10^-4^ | 1 | 873 | 9.27×10^14^ | 0.15 | 1.11 |
| 6.5×10^-4^ | 0.1 | 573 | 3.46×10^14^ | 0.17 | 3.56 |
| 6.5×10^-5^ | 1 | 873 | 2.96×10^14^ | 0.27 | 1.97 |
| 6.5×10^-3^ | 1 | 1000 | 1.67×10^14^ | 0.90 | 5.15 |
| 7.7×10^-5^ | 10 | 873 | 5.43×10^13^ | 1.77 | 13.0 |
| 6.5×10^-3^ | 1 | 1173 | 3.27×10^13^ | 5.24 | 23.12 |
| 6.5×10^-4^ | 1 | 1173 | 1.03×10^13^ | 9.32 | 41.11 |
| 6.5×10^-3^ | 0.1 | 873 | 6.37×10^13^ | 18.30 | 134.40 |
| 6.5×10^-5^ | 0.1 | 873 | 6.44×10^12^ | 57.54 | 422.60 |
| 6.5×10^-4^ | 0.05 | 873 | 9.70×10^12^ | 132.61 | 973.95 |
| 6.5×10^-5^ | 0.05 | 873 | 3.12×10^12^ | 233.81 | 1717.30 |
| 6.5×10^-4^ | 0.1 | 1173 | 4.20×10^12^ | 462.23 | 2038.72 |
| 6.5×10^-5^ | 0.1 | 1173 | 9.30×10^11^ | 982.30 | 4332.53 |

**Physical meaning of the parameter λ**


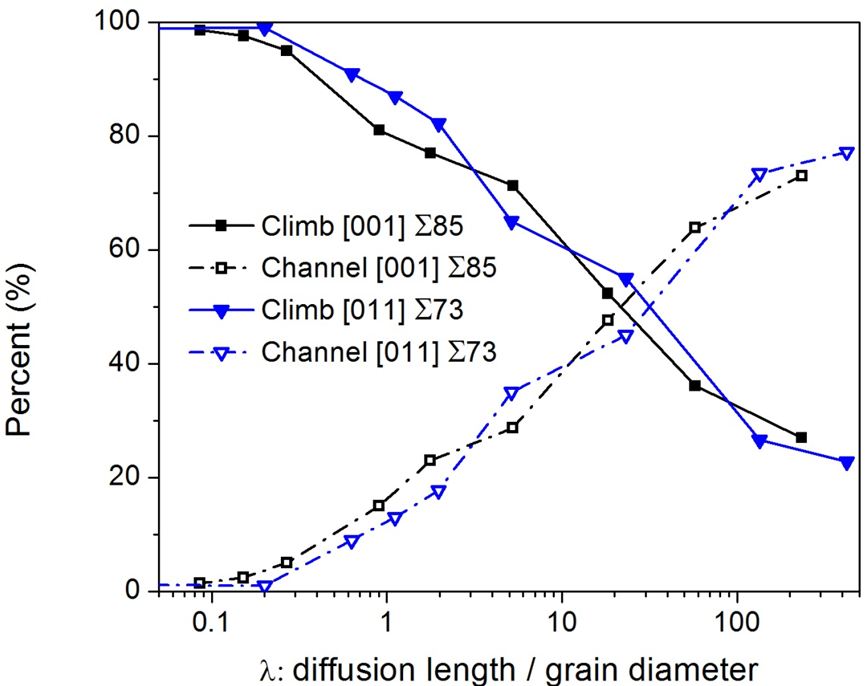


**Figure S6** | The roles of [001] Σ85 tilt GB and [011] Σ73 tilt GB in annihilating defects under various irradiation conditions. The ratio of C*_i_* flux to Si*_i_* flux is set as 100. Climb means annealing of defects at existing jogs or jog nucleation. Channel means diffusion of defects along GBs to other sinks.

In the main text we introduced a paramater λ, defined as the diffusion distance divided by the dislocation length. Here, we show that λ is a universal parameter that can describe a transition from diffusion to climbing regimes in all GBs.

We perform simulations with the dislocation line model for 2 specific STGBs: [001] Σ85 and [011] Σ73. These two GBs are chosen because of the difference in migration barriers of interstitials between them (1.1 eV in [001] Σ85 vs. 0.8 eV in [011] Σ73). Though they are both higher than the bulk diffusion barrier (0.74 eV), the difference in migration barriers between the two GBs may predict different roles of GBs in annihilating defects under the same irradiation condition. The percenetages of interstitials annihilated by climbing and diffusion are plotted as a function of diffusion length divided by dislocation length in Figure S6. Both GBs show the same trend in percentages of interstitials in climbing and diffusion channel regimes with respect to the parameter λ. This implies the generality of the parameter λ in predicting kinetics of defect diffusion in GBs.

Although the the value of the parameter λ for the transition between diffusion and climbing regimes does not depend on the GB, the irradiation conditions that produce a given value of λ will differ between the GBs. For example, to obtain a value of λ = 0.90 in a grain with the diameter of 1 μm under a dose rate of 6.5×10^-3^ dpa/s, [001] Σ85 requires the irradiation temperature to be 1000 K whereas for [011] Σ73 this temperature is 873 K (Table S2). This is because of the difference in migration barriers of interstitials between these two GBs. Therefore, when translating the parameter λ into the experimental conditions, we have to examine each specific GB case by case.

**Supplementary video 1** | Simulation of the dislocation line model for SiC with grain diameter of 100 nm irradiated at 573 K under dose rate of 6.5×10^-3^ dpa/s to a total dose of 5 dpa. Red spheres represent the dislocation core, and the blue line indicates the position of the dislocation line before irradiation.

**References**

1 Gao, F. & Weber, W. J. Empirical potential approach for defect properties in 3C-SiC. *Nucl. Instrum. Meth. B* **191**, 504-508 (2002).

2 Bockstedte, M., Mattausch, A. & Pankratov, O. Ab initio study of the migration of intrinsic defects in 3 C− SiC. *Phys. Rev. B* **68**, 205201 (2003).

3 Kresse, G. & Joubert, D. From ultrasoft pseudopotentials to the projector augmented-wave method. *Phys. Rev. B* **59**, 1758 (1999).

4 Henkelman, G., Uberuaga, B. P. & Jónsson, H. A climbing image nudged elastic band method for finding saddle points and minimum energy paths. *J. Chem. Phys.* **113**, 9901-9904 (2000).

5 Shrader, D., Khalil S., Gerczak T., Allen T., Heim A., Szlufarska I., Morgan D. Ag diffusion in cubic silicon carbide. *J. Nucl. Materials* **408**, 257-271 (2011).
