# Supplementary figures and images for: The Multiple Roles of Small-Angle Tilt Grain Boundaries in Annihilating Radiation Damage in SiC
Source: Sci Rep. 2017 Feb 9;7:42358. doi: 10.1038/srep42358 (PMC5299421; doi:10.1038/srep42358)

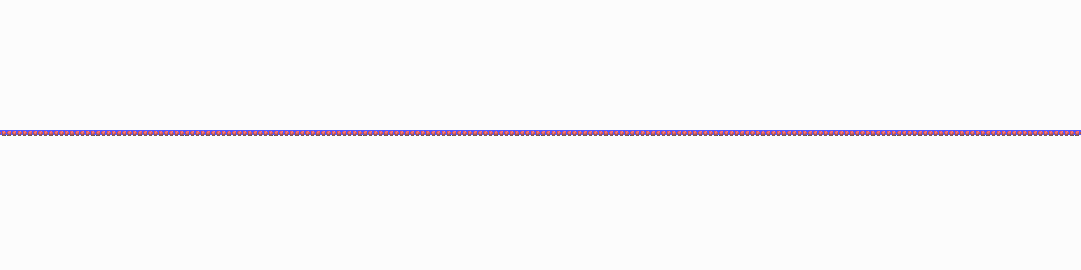

Supplement: Supplementary Video 1 [file srep42358-s2.gif]
